# A case of ureteral myeloid sarcoma post-renal transplantation

**DOI:** 10.1186/s12882-018-0838-0

**Published:** 2018-02-27

**Authors:** Liang Ying, Lei Tian, Yuanyuan Xie, Qin Wang, Xiajing Che, Jiayi Yan, Lijing Shen, Honghui Huang, Fangyuan Chen, Ming Zhang, Zhaohui Ni, Shan Mou

**Affiliations:** 1 Department of Nephrology, Molecular Cell Laboratory for Kidney Disease, Renji Hospital, School of Medicine, Shanghai Center for Peritoneal Dialysis Research, Shanghai Jiaotong University, Shanghai, 200127 People’s Republic of China; 2 Department of Hematology, Renji Hospital, School of Medicine, Shanghai Jiaotong University, Shanghai, People’s Republic of China; 3 Department of Urology, Renji Hospital, School of Medicine, Shanghai Jiaotong University, Shanghai, People’s Republic of China

**Keywords:** Myeloid sarcoma, Renal transplantation, Acute myeloid leukaemia

## Abstract

**Background:**

Significant attention has been directed toward the high incidence of malignant tumours that occur post-transplantation. However, there are few reports of myeloid sarcomas (MSs) following renal transplantation.

**Case presentation:**

This case report describes a 26-year-old male patient who presented with repeatedly high creatinine levels and hydronephrosis six months post-renal transplantation. Surgical pathology revealed ureteral MS; however, the tumour recurred following resection. Bone marrow biopsy indicated that the patient also had acute promyelocytic leukaemia. The tumour was treated with local radiotherapy, and the leukaemia was treated with systemic chemotherapy. The patient’s conditions were satisfactory at the one-year follow-up.

**Conclusions:**

This report is the first to describe a ureteral MS post-renal transplantation. Our findings suggest that surgical resection combined with radiotherapy and chemotherapy can help control the status of patients with this condition.

## Background

Lymphoproliferative diseases that occur post-renal transplantation have been well described. In contrast, few studies have reported cases of myeloid sarcoma (MS). Notably, MS can be misdiagnosed as other diseases. Cases of MS post-renal transplantation are rare, and not a single case has been reported in a transplanted kidney or transplanted area of skin. This case report describes the first case of ureteral MS post-renal transplantation.

## Case presentation

The patient was a 26-year-old male with end-stage renal disease caused by primary glomerular disease. He had undergone regular haemodialysis for more than 7 years. The donor was a 21-year-old female who had died from a cerebral haemorrhage. The donated kidney was healthy (type A blood, panel-reactive antibody (PRA) type I, 0% and type II, 0%). The patient underwent induction therapy with methylprednisolone and anti-thymocyte globulin and was maintained on prednisone (7.5 mg daily), Myfortic (720 mg daily) and tacrolimus (3 mg daily). His renal function recovery was satisfactory after surgery (serum creatinine (sCr) 100 μmol/L). A postoperative Doppler ultrasound examination of the transplanted kidney indicated that the size of the transplanted kidney was 112 × 40 mm, and the measurement of hydronephrosis that can be quantified as the diameter before and after the renal pelvis separation was 5 mm.

**Fig. 1:**
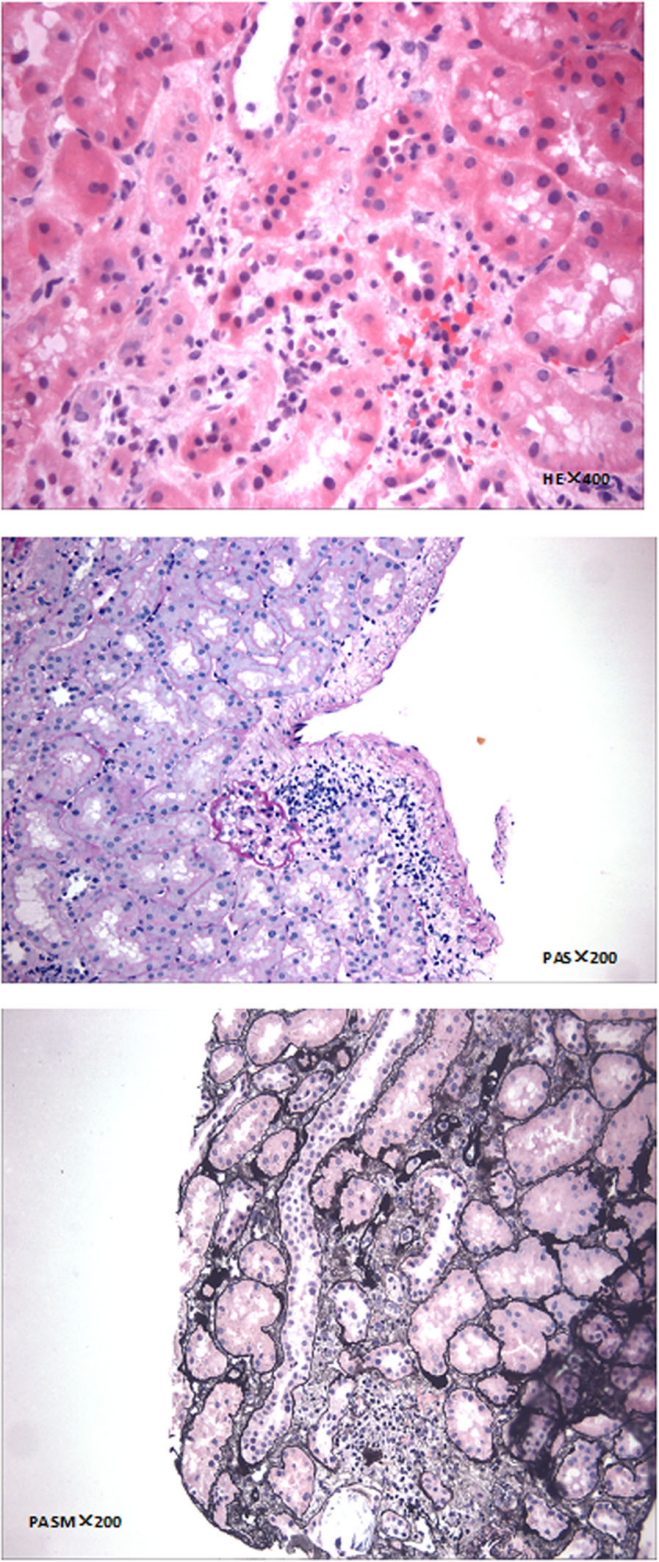
Renal biopsy pathology indicated compliance with Banff borderline changes

Five months after the transplantation surgery, no symptoms of discomfort were observed, and the patient’s urine volume was normal. Biochemical analyses indicated that the sCr level was elevated (240 μmol/L). The tacrolimus concentration was 5.3 ng/mL. No abnormalities were identified in the whole blood analysis. According to the Doppler ultrasound examination of the transplanted kidney, the size of the transplanted kidney was 115 × 42 mm, the measurement of hydronephrosis was 7 mm, and the resistance index was 0.7. A puncture biopsy of the transplanted kidney was performed, and the pathological results were consistent with Banff borderline changes that were characterized by mild tubulitis, interstitial inflammation, and no intimal arteritis (Fig. 1). In consideration of the acute rejection of the transplanted kidney, pulsed high-dose steroid therapy was initiated, and the sCr level subsequently decreased (150 μmol/L). Doppler ultrasound examination of the transplanted kidney was performed again, and the results indicated that the size of the transplanted kidney was 118 × 44 mm, the measurement of hydronephrosis was 10 mm, and the resistance index was 0.7. After one week, although no symptoms of discomfort were observed, the urine volume was reduced, and the sCr level was elevated again (351.2 μmol/L). No abnormalities were observed in the whole blood analysis. Doppler ultrasound examination of the transplanted kidney revealed that the size of the transplanted kidney was 140 × 61 mm, the measurement of hydronephrosis was 27 mm, and the resistance index was 0.68. Therefore, a double J (D-J) stent was placed retrogradely into the allograft ureter using a ureteroscope. Moreover, the mucosa seen through the ureteroscope was of normal integrity. After the operation, the urine volume increased, and the renal function recovered (sCr 130 μmol/L). Doppler ultrasound examination of the transplanted kidney demonstrated that the size of the transplanted kidney was 135 × 51 mm, and the measurement of hydronephrosis was 15 mm.

**Fig. 2:**
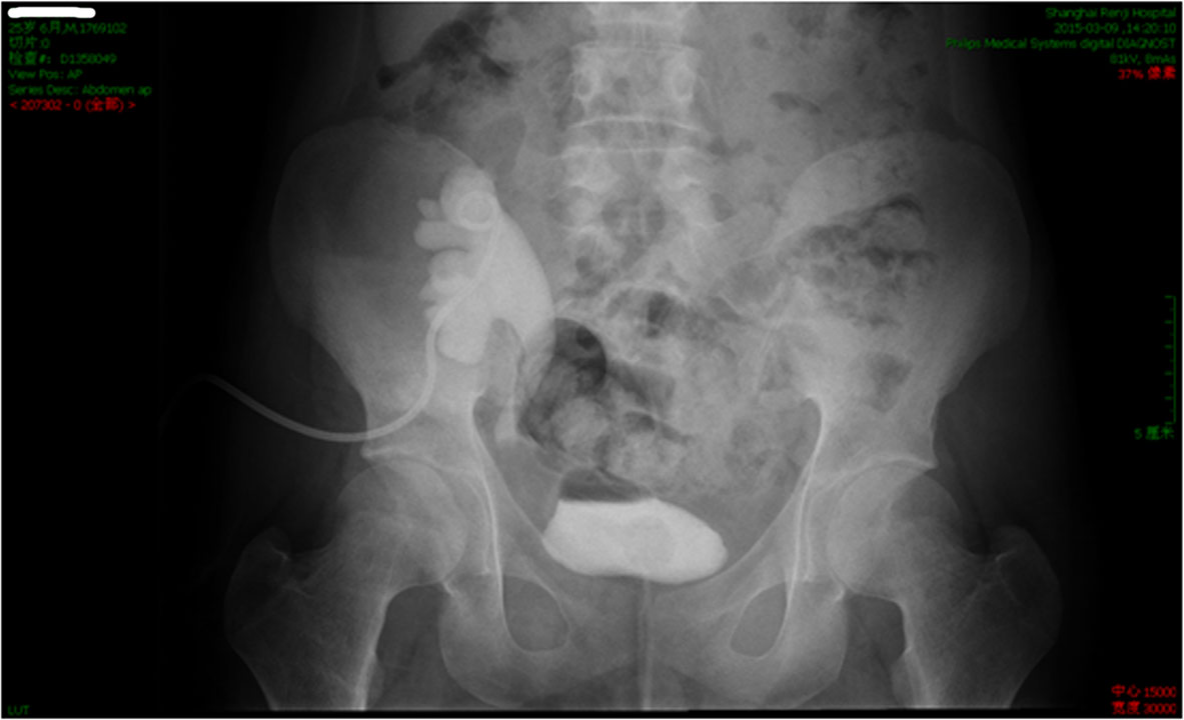
Urinary antigrade pyelogram revealing stenosis of distal allograft ureter

**Fig. 3:**
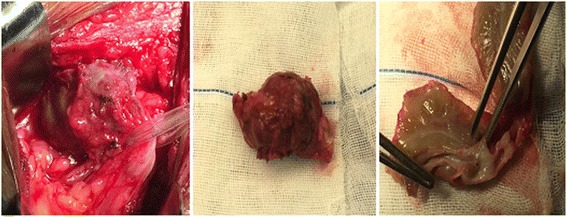
Surgical pictures showing the encapsulated mass on the end of the allograft ureter before and after excision. The texture was hard, and the lesion encapsulated

Eight weeks after D-J tube catheterization, reduced urine volume and increased sCr (310 μmol/L) were again observed. No abnormalities were observed in the whole blood analysis. Replacement of D-J tube failed, and percutaneous nephrostomy (PCN) was performed for the transplanted kidney. In the operation, a 9 French (F) PCN was placed by interventional radiology. The patient’s urine volume increased, and his renal function recovered (sCr 89 μmol/L). Urinary pyelogram with antegrade contrast was performed, and stenosis was found at the end of the ureter (Fig. 2). Accordingly, exploratory laparotomy and replantation of the transplanted kidney’s ureter and bladder were performed. During surgery, an encapsulated mass was observed at the end of the allograft ureter. The mass was dissected out and an encapsulated mass was observed at the end of the ureter. The texture of the mass was hard and the envelope was complete. Following resection of the mass involving the distal allograft ureter, a modified Lich-Gregoir ureteroneocystomy was preformed over an indwelling D-J stent. The surgical observations are displayed in Fig. 3. The following pathological diagnosis was made: a “ureteral neoplasm post-renal transplantation” malignant small cell tumour with a tendency to develop into MS. The immunohistochemical diagnosis was as follows: “ureteral neoplasm post-renal transplantation” with proliferative tumour cells, LCA(−), CD10(−), CD3(−), CD5(−), CD79a(−), Bcl-2(+), PAX-5(−), Ki67(60%+), CD20(−), CD21(−), CD23(−), CD138(−), MUM-1(−), κ(−), λ(−), CyclinD1(−), CK(−), Vim(+), MPO(+), CD56(−), TdT(−), SMA(+), PG-M1(−), HMB45(−), CD99(+), and TIA-1(−). The lump was confirmed as a malignant small cell tumour with a tendency to develop into MS. The pathological diagnosis made at the cancer hospital was a ureteral neoplasm post-renal transplantation. Based on the haematoxylin and eosin (H&E) and immunohistochemistry analyses the resected mass presented a small cell anaplastic MS. Postoperative micturition was unobstructed. The PCN and D-J tubes were removed, and the renal function was stable.

More than four months after the lump was resected, the patient’s urine volume progressively decreased, and his sCr level increased to 303 μmol/L. No abnormalities were observed in the whole blood analysis. Computed tomography (CT) indicated hydronephrosis in the transplanted kidney. After catheterization of the transplanted kidney, increased thickness was observed on positron emission tomography (PET)-CT in part of the ureter accompanied by slightly increased FDG metabolism; thus, a tumour recurrence was diagnosed. Increased FDG metabolism of the skin and subcutaneous and soft tissues associated with the surgical approach in the right inferior abdominal wall was identified in the pelvic cavity, bilateral femur, head and face. It was impossible to exclude the possibility of a tumour. No abnormalities were identified in the bone marrow biopsies from two different sites. The planned radiotherapy isodose to the ureter was 26 Gy in 13 fractions; however, the patient only received 11 fractions for a total dose of 22 Gy. The urine volume returned to normal, and the sCr level remained stable at approximately 90 μmol/L. A third bone marrow biopsy was performed after one month (from the posterior upper spine of the right ilium) and yielded the following results: decreased bone marrow hyperplasia; megakaryocytes present; granulocytes accounted for 53.5% of cells and were observed in all stages; red blood cells accounted for 36.5% of the cells and were mainly middle, advanced and young red blood cells; mature lymphocytes accounted for 8.5% of the cells; and the biopsy was positive for promyelocytic leukaemia (PML) and positive for the retinoic acid receptor alpha (RARα). Karyotype analysis revealed 46 and XY [20]. In the blood tumour immune analysis, the percentage of lymphocytes was reduced, and the percentage of granulocytes was increased. No significant abnormalities were observed in the immunophenotype. The diagnosis was acute promyelocytic leukaemia. Arsenicals plus all-trans retinoic acid (ATRA) chemotherapy was administered, the tacrolimus capsule dosage was reduced, and rapamycin was used for anti-rejection treatment. To date, the patient has undergone 6 cycles of chemotherapy. His urine volume is normal and his renal function is stable (sCr 80–100 μmol/L).

## Discussion

Malignant tumours are a common complication after renal transplantation. Among subjects who undergo renal transplantation, 9%–18% die of cancer, and the overall risk of post-transplantation malignant tumours is 3–5 times higher than that of control groups matched for age, regardless of pre-transplantation dialysis [1, 2]. Post-transplantation tumours, which are related to the application of immune preparations and extensive viral infections, are diverse and can occur in multiple organ systems [3, 4].

MSs are extramedullary limited tumours composed of undifferentiated cells originating from the medullary system and are is often accompanied by acute myeloid leukaemia (AML), which can occur in any part of the body, although it primarily occurs in the skin, soft tissues, skeleton and lymph nodes (a rare solid tumour) [5]. Myeloid neoplasms post-organ transplantation are rare. Compared with renal transplantation, MS occurs more frequently after heart or lung transplantation, which might be related to DNA damage resulting from prolonged exposure to immunosuppressive drugs [6]. Although MS can occur at any site, this disease rarely occurs in the ureter, and this report is the first to describe a case of ureteral MS post-renal transplantation.

MS symptoms are usually secondary to the space-occupying effect of the tumour, which can result in misdiagnosis and missed diagnosis. The patient in the current report repeatedly presented with post-renal obstruction, which resulted in repeatedly elevated sCr levels. After the obstruction was relieved, the sCr level decreased, and the surgical pathology indicated MS. Immunohistochemical testing plays an important role in the diagnosis of MS. Therapies for MS generally include local radiotherapy, systemic chemotherapy, immunotherapy, surgery and combined treatment. Moreover, MS is commonly an early manifestation of AML; it has been reported that 71% of MS patients who do not receive anti-leukaemia chemotherapy develop AML within a median of 7 months [7]. Local radiotherapy is effective for local lesions without leukaemia, but it cannot prevent the progression of leukaemia. After surgical resection, the current patient suffered from the recurrence of local MS lesions, and his condition was relieved through local radiotherapy. Multiple bone marrow biopsies indicated AML; therefore, systemic chemotherapy was administered for more than six months. To date, no recurrence has occurred, and the patient’s renal function has remained stable.

MSs are rare malignant tumours that occur after renal transplantation. Al Ghunaim et al. reported the first case of post-renal transplantation MS, which was limited to the kidney and perirenal areas [8]. Palanisamy et al. reported that a patient developed MS from the organ donor, and in two subjects with renal transplantation, the MSs were limited to the transplanted kidney [9]. Additionally, a study by Shen et al. from China reported a case of MS in a transplanted kidney that was complicated by multiple skin and subcutaneous MSs [10]. No leukaemia was found in the MS patients in these reports. Indeed, the current patient represents the first reported case of ureteral MS post-renal transplantation in the transplanted kidney accompanied by AML. Neither the donor nor the recipient had a history of tumours. Six months post-transplantation, MS was observed accompanied by AML. Through a combination of surgery, local radiotherapy and systemic chemotherapy, the patient’s condition was controlled.

## Conclusion

Although ureteral post-renal transplantation MS rarely occurs, an immediate and accurate diagnosis is important. Specifically, our findings suggest that surgical resection combined with radiotherapy and chemotherapy can help control patients’ condition. Further studies are needed to investigate the clinical features and pathogenesis of this condition; meanwhile, multidisciplinary diagnosis and treatment.

## Abbreviations

AML: Acute myeloid leukaemia
MS: Myeloid sarcoma
PCN: Percutaneous nephrostomy
sCr: Serum creatinine
